# Portable respiratory polygraphy monitoring of obese mothers the first night after caesarean section with bupivacaine/morphine/fentanyl spinal anaesthesia

**DOI:** 10.12688/f1000research.13206.2

**Published:** 2018-02-07

**Authors:** Anette Hein, Jan G. Jakobsson

**Affiliations:** 1 Anaesthesia & Intensive Care, Institution for Clinical Sciences, Karolinska Institutet, Danderyds University Hospital, Stockholm, Sweden

**Keywords:** Caesarean section, obesity, intrathecal morphine, respiration, respiratory depression, apnoea/hypopnea

## Abstract

**Background: **Obesity, abdominal surgery, and intrathecal opioids are all factors associated with a risk for respiratory compromise. The aim of this explorative trial was to study the apnoea/hypopnea index 1st postoperative night in obese mothers having had caesarean section (CS) in spinal anaesthesia with a combination of bupivacaine/morphine and fentanyl.

**Methods: **Consecutive obese (BMI >30 kg/m 2) mothers, ≥18 years, scheduled for CS with bupivacaine/morphine/fentanyl spinal anaesthesia were monitored with a portable polygraphy device Embletta /NOX on 1 ^st^ postoperative night. The apnoea/hypopnea index (AHI) was identified by clinical algorithm and assessed in accordance to general guidelines; number of apnoea/hypopnea episodes per hour: <5 “normal”, ≥5 and <15 *mild sleep apnoea*, ≥15 and <30 *moderate sleep apnoea,* ≥ 30 *severe sleep apnoea*. Oxygen desaturation events were in similar manner calculated per hour as oxygen desaturation index (ODI).

**Results: **Forty mothers were invited to participate: 27 consented, 23 were included, but polysomnography registration failed in 3. Among the 20 mothers studied: 11 had an AHI <5 ( *normal*), 7 mothers had AHI ≥5 but <15 ( *mild OSAS*) and 2 mothers had AHI ≥15 ( *moderate OSA*), none had an AHI ≥ 30. The ODI was on average 4.4, and eight patients had an ODI >5. Mothers with a high AHI (15.3 and 18.2) did not show high ODI. Mean saturation was 94% (91-96%), and four mothers had mean SpO _2_ 90-94%, none had a mean SpO2 <90%.

**Conclusion: **Respiratory polygraphy 1 ^st^ night after caesarean section in spinal anaesthesia with morphine in moderately obese mothers showed AHIs that in sleep medicine terms are considered normal, mild and moderate. Obstructive events and episodes of desaturation were commonly not synchronised. Further studies looking at preoperative screening for sleep apnoea in obese mothers are warranted but early postop respiratory polygraphy recording is cumbersome and provided sparse important information.

## Introduction

In caesarean section (CS), intrathecal morphine (ITM) is associated with better postoperative course with less pain and shorter time to mobilisation ^1^. Respiratory depression associated with morphine is well known side effect. The µ-receptor has been suggested to be the key target for the respiratory depressant effect of morphine and further the sophisticated neuronal interaction in the ventral part of the brain stem ^2^. Respiratory depression is the most feared adverse effect from intrathecal opioids, and although rare (0–0.9%), it is the reason for some anaesthesia units to withhold the addition of morphine to spinal anaesthesia for CS ^3– 5^. It has been suggested that ITM in doses <0.3 mg (300 µg) is associated with a lower risk of respiratory depression compared with doses >0.3 mg, and a tendency to a lower risk as compared to systemic opioids ^3, 6^. No analgesic benefit and increased incidence and severity of side effects for doses exceeding 100 µg intrathecal morphine for CS spinal anaesthesia have been described ^1^.

Respiratory depression is not well defined and there is still not a general definition of respiratory depression to opioids ^7^. A recent meta-analysis around risks for respiratory depression commented that multiple definitions were used in the included studies ^8^. Similarly, a review concerning the “definitions of respiratory depression”, explicitly addressing ITM, found no common description ^9^. They also clearly commented the need for further research for defining what is a clinically significant respiratory impairment caused by ITM and how it best can be monitored ^9^. It has been proposed to define respiratory depression associated to opioids solely as a respiratory rate (RR) of <10 breaths per minute ^10^.

There are several factors that can contribute to postoperative respiratory depression. Residual effects from anaesthesia, abdominal surgery, obesity, immobilisation and opioid analgesics may all contribute to its occurrence ^7, 11^. Postoperative respiratory depression after CS, performed in spinal anaesthesia including neuroaxial morphine, is found in low frequency (0/5036, 8/856, 6/1915) ^4, 12, 13^. Studies explicitly assessing the risk of respiratory compromise in obese patients, i.e. mothers with a high BMI (Body Mass Index) are sparse. No respiratory event, with need for naloxone, defined as RR ≤8 breaths/min, oxygen saturation <90% or Richmond Agitation Sedation Scale <-2, was found in a retrospective study of 5036 mothers who had a CS with neuroaxial morphine, where 63% of patients were obese (BMI > 30 kg/m ^2^), and the most commonly used morphine dose was 3 mg epidural and 0.15 mg spinal ^4^. A study in women having intrathecal addition of 0.2 mg morphine in spinal analgesia for CS found 8/856 cases of respiratory depression, all eight cases were obese and naloxone treatment did not reverse the respiratory depression during sleep ^12^. A review published in 2008 addressing the risk, monitoring and prevention of respiratory depression associated with neuroaxial morphine in the obstetric setting supports the need for observation up to 24 hours following neuraxial morphine, due to the duration of the depressed CO _2_ sensitivity ^3^. This paper also commented on the present lack of efficient, simple and mother-friendly monitoring equipment; but support the monitoring of RR, and sedation monitoring in healthy mothers with addition of oxygen saturation in obese ^3^.

Portable sleep test equipment so called at-home polygraphy monitors are commonly used for screening for obstructive sleep apnoea. This equipment assesses respiration; oro-nasal actual gas flow, thoracic and abdominal movements and saturation. The registration is analysed and processed in indices: apnoea-hypopnea index (AHI) and oxygen desaturation index (ODI) using defined algorithms.

The aim of this explorative trial was to study the apnoea/hypopnea index the 1st postoperative night in obese mothers having had caesarean section in spinal anaesthesia with a combination of bupivacaine/morphine and fentanyl. Our hypothesis was that the combination of risks, obesity, intrathecal opioids, and 1st postoperative night should show a high incidence of AHIs of more than 30.

## Methods

### Ethical statement

The study was approved by the Regional Ethical Review Board in Stockholm (2015/1257-31/2).

### Patients

A prospective postoperative observational study was conducted from December 2015 to October 2017. Parturients with BMI >30 kg/m ^2^ at the first antenatal consultation, who were planned for elective CS with low transverse incision under spinal anaesthesia, were included in the study after informed verbal and written consent.

Patients with language difficulties and known diagnosed obstructive sleep apnoea (OSA) receiving treatment, as those with continuous positive airway pressure or mouth guard, or any known contraindication to ITM were excluded.

Patients were informed at the preanaesthetic consultation about spinal analgesia and that they would receive a mixture of local anaesthesia, bupivacaine, and two opioids, fentanyl and morphine, in order to optimize perioperative and postoperative anaesthesia, according to the standard routine at our department (Anaesthesia & Intensive Care Unit, Danderyd Hospital).

### Spinal anaesthesia procedure

Spinal anaesthesia was performed with the patient in sitting or left lateral position, at the anaesthetists’ preference, using a 25-gauge pencil-point needle. All patients received a mixture of heavy bupivacaine (11–12 mg), fentanyl (10 µg) and morphine (100 µg), per standard routines for CS in our department.

The mothers received preoperative 1.5 g paracetamol orally, and paracetamol was continued postoperatively 1 g every six hours or 1.330g every eight hours. Ibuprofen (400 mg) was administered every eight hours. A further rescue therapy for the handling of pain, nausea and vomiting or pruritus was administered per the department’s routines.

Routine monitoring in the postoperative and obstetric ward after spinal anaesthesia includes the following: checking for sedation, and if sedated counting RR every hour; pain by NRS/VAS; heart rate and blood pressure; control of bleeding; urine output; mobilization; and breast feeding. First mobilization, to stand by the bed, is usually encouraged at about 5–6 hours postoperatively. Urine catheter is normally removed after the first postoperative night.

### Data collection

Patients were informed at the preanaesthetic consultation about extended postoperative monitoring in addition to routine monitoring: nasal catheter to measure expiration flow; finger probe to measure oxygen saturation; thoracic and abdominal strings to collect breathing movements for polygraphy registration; a portable OSA breathing pattern monitor, Embletta (ResMed Sweden AB, Kista, Sweden)/Nox Sleep monitor (Nox Medical, Iceland); and a combined ear-probe for transcutaneous carbon dioxide (TcCO _2_)/oxygen saturation (SpO _2_) monitor (Tosca Radiometer Medical ApS, Denmark).

The respiratory polygraphy data was stored in the equipment and further analysed with a standard analysis program (ResMed Sweden AB, Kista, Sweden). The equipment software is screening for both the AHI and ODI ^14, 15^. The program also provides each desaturation episode minimum blood oxygen saturation SpO2 level measured, *the oxygen desaturation episode nadir*.

**Apnoea** was classified in accordance to the American Academy of Sleep Medicine (AASM) as a drop in the polygraphy peak signal excursion by ≥ 90% of pre-event baseline air-flow signal ^16^. The breathing disturbance was classified as mild AHI ≥5 and <15, moderate ≥15–<30 and severe ≥30. The duration of the ≥90% drop in sensor signal must be ≥10 seconds ^16^. **Hypopnea** was classified by as a drop in the peak signal excursion by ≥30% of pre-event baseline ^16^. The duration of the ≥30% drop in signal excursions must be ≥10 seconds ^16^.

Respiratory monitoring, respiratory polygraphy and Tosca registration was in the late afternoon - evening of the first postoperative and continued during night. For the 3–5 first hours postpartum, the patients were observed awake in in accordance to routines in the postoperative department.

All patients answered a standardised ESS ( Epworth Sleepiness Scale) questionnaire at time of enrolment. With grading ^1^; 0–5 Lower Normal Daytime Sleepiness, 6–10 Higher Normal Daytime Sleepiness, 11–12 Mild Excessive Daytime Sleepiness, 13–15 Moderate Excessive Daytime Sleepiness, 16–24 Severe Excessive Daytime Sleepiness.

### Statistical analysis

Data is presented as the mean and standard deviation; categorical data are presented as frequencies. The study is explorative and observational; thus, no power analysis has been conducted. Differences were tested with Student’s t-test for continuous variables and Chi-squared test for categorical data. P<0.05 was considered significant. Data was analysed with StatView (v1.04) for MAC.

## Results

**Figure 1.**
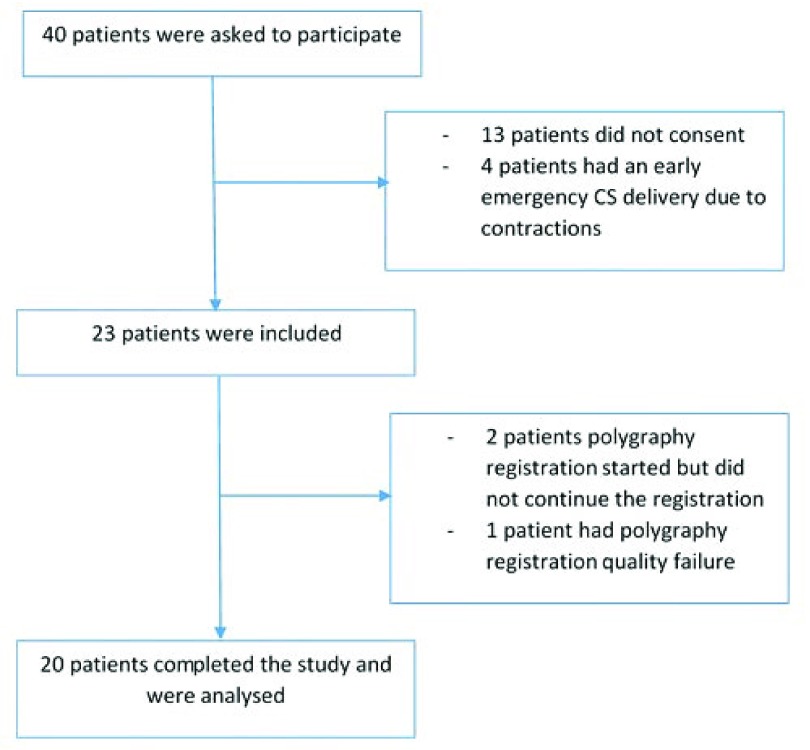
Patient inclusion, polygraphy first night after CS in spinal anaesthesia with ITM in obese mothers.

Forty mothers were invited to participate: 27 mothers consented but four of them had an early emergency CS delivery due to contractions, thus 23 mothers were included, but polygraphy registration failed in 3 (see Figure 1). Therefore, 20 mothers were included in analysis.

In all 20 mothers, mean age 35 ± 5 (24–43) years, mean BMI 35 ± 4 (30–42), and mean ESS 6 ± 3 (0–12) were studied.

Five mothers had an ESS of <5, 12 had an ESS score between 5 and 10, and 3 scored >10 on the preoperative ESS screening.

All surgery and anaesthesia was uncomplicated.

Mean bed time during the polygraphic registration was 585 minutes (378–818).

Mean AHI was 6.6 ± 5.2 (0–18.2) and mean ODI was 4.4 ± 3 (0–10.3). A total of 11 mothers had “normal” (<5) AHI, 7 had an AHI between 5 and 15, and 2 had an AHI 15–30 (15.3 and 18.2). No mothers had an AHI ≥30. The longest apnoea duration was (mean) 30 ± 27 seconds, and mean longest hypopnea duration 55 ± 25 seconds.

Mean saturation was 94% (91–96%) and four mothers had mean saturation between 90 and 94%, but no had a mean SpO2 < 90%. Nadir, lowest saturation registered, was in mean 71% (49 – 81%).

In total, 11 mothers had an ODI <5, 8 had ODI between 5 to 10, and 1 mother had an ODI of 10.3. The 2 high AHI (15.3 and 18.2) mothers did not show high ODI or signs of hypercapnia on the transcutaneous CO _2_ registration.

Mean TcCO _2_ was 4.7 ± 0.3 (4.1–5.2) kPa, and mean of max TcCO _2_ was 5 ± 0.5 kPa. There were no TcCO _2_ >5.9 kPa.

**Figure 2.**
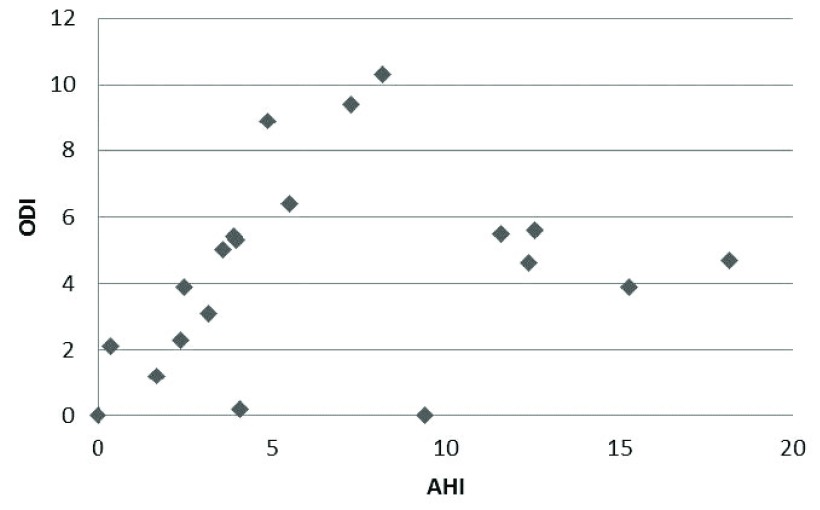
The apnoea-hypopnea index (AHI) and oxygen desaturation index (ODI) measures plotted for each mother.

The pattern between AHI, ODI, BMI and ESS was overall scattered without correlation; Figure 2 describes the AHI and ODI pattern.

None of the mothers showed clinical signs or symptoms of severe respiratory depression as assessed by routine clinical monitoring. Six mothers required rescue buprenorphine oral 0.2 – 0.4 mg and one mother had 5 mg intravenous morphine for postoperative pain.

Raw data for the study polygraphy on the first night after caesarean section in spinal anaesthesia with morphine in obese mothers by Hein *et al.* The three patients who were excluded from analysis are highlighted Click here for additional data file. Copyright: © 2018 Hein A and Jakobsson JG 2018 Data associated with the article are available under the terms of the Creative Commons Zero "No rights reserved" data waiver (CC0 1.0 Public domain dedication).

## Discussion

We did not find any AHI defined as severe sleep apnoea in this at risk cohort; obese mothers 1st night after caesarean section in spinal anaesthesia including morphine and fentanyl. Two out of 20 mothers included in the present study had an AHI in the lower range of moderate sleep apnoea (15.3 and 18.2). Preoperative screening and testing for sleep apnea in accordance to guidelines is of course of value also mild/moderate sleep apnea may cause adverse effects ^17^ but early postoperative respiratory polygraphy was found cumbersome and seems not to provide much critical information. Mothers with a high AHI did not show typical high oxygen desaturation index or TcCO _2_ elevation. We cannot give any firm explanation to the discrepancy between the AHI and the ODI. Indeed, we did see frequent short episodes of oxygen saturation decrease, but we are unfortunately not able to assess whether these events were related to bradypnea or shallow breathing. We did not register any increase in TcCO _2_. The TcCO _2_ monitoring had a 5-minute averaging algorithm, thus it was not set for the detection of brief episodes of CO _2_ elevation. Respiratory depression typically progresses slowly ^3^.

Studies with polygraphy registration sleep apnoea signs, associated with ITM are sparsely performed. The effects of 30 mg oral morphine on patients with mild to moderate obstructive sleep apnoea has been investigated previously ^18^. This study showed that morphine may paradoxically improve sleep apnoea ^18^. Similarly, a study of the effects of remifentanil infusion, and found a decrease in obstructive events, but a worsening in oxygenation during the infusion ^19^. Cole *et al.* studied the respiratory effects of ITM (dose, 300 µg) in prospective randomized fashion among patients having knee replacement in spinal anaesthesia ^20^. They found similarly to the present study that night time respiratory polygraphy is cumbersome and associated with high patient non-compliance ^20^. Among the patients studied in that study, the incidence of apnoea and hypopnea episodes was not significantly different compared to the control group of patients that did not receive ITM ^20^. Median mean oxygen saturation was, however, significantly lower among the ITM patients and the occurrence of “mild and moderate hypoxia” was also high in the ITM group. In another previous study, 45 obese patients undergoing elective bariatric surgery with general anaesthesia were monitored in a similar fashion with portable polygraphy equipment during the first postoperative night on the general ward; only two patients with an AHI >5 and only three with an ODI >5 was found ^21^. Therefore, our findings, that registration during the first postoperative night following CS with spinal anaesthesia with a modest dose of morphine in obese mothers did not show any high incidence of AHI and ODI, might not be that surprising. It is also in line with a Cochrane review assessing the effects of opioid, hypnotic and sedating medications on sleep-disordered breathing in adults with obstructive sleep apnoea ^22^. None of the studied drugs in the Cochrane review produced a significant increase in AHI or ODI and two trials have shown a beneficial effect on OSA ^22^.

However, a recent paper *,* catastrophic events in patients related to obesity and sleep apnoea, stating that ‘Morbid obesity, male sex, undiagnosed OSA, partially treated/untreated OSA, opioids, sedatives, and lack of monitoring are risk factors for death or near-death events’ ^23^. Preoperative assessment for sleep apnea should of course be performed eventually including polysomnography in at risk patient ^^24, 25^^. For pregnant obese women, especially in case of hypertension, snoring and/or gestation diabetes, that are associated with increased incidence of OSA, assessment seems warranted already during pregnancy since untreated OSA may affect maternal and foetal outcome ^24,26, 27^.

We cannot further comment on how and how long obese patients having ITM should be monitored. It seems still of importance to monitor respiration in patients at risk. Monitoring of AHI and ODI during the early postoperative period seems, however, not to be of major help. It may be that simple RR monitoring, TcCO _2_ measure and SpO _2_ are more feasible techniques ^28, 29^. Kopka *et al.* suggest that TcCO _2_ may be more effective in detecting respiratory depression compared to SpO _2_ when patients receive supplementary oxygen ^30^. Ladha *et al.* studied oximetry after CS having 150 µg morphine intrathecal ^31^. They found frequent mild desaturation events and increased risk in patients with obesity. Bauchat *et al.* studied TcCO _2_ tension and found that hypercapnia events (>50 mm Hg for ≥2-minute duration) occurred frequently in women receiving 150 μg ITM for post-caesarean analgesia; higher baseline TcCO _2_ readings were observed in women who had hypercapnia events ^32^. Dalchow *et al.* studied both TcCO _2_ and SpO _2_ and found more frequent changes, hypercapnia as compared to desaturation ^33^. They concluded, ‘The incidence of opioid-induced respiratory depression detected by TOSCA is higher than previously reported by other monitoring methods. TOSCA may have a role in detecting subclinical respiratory depression in the obstetric population. Further studies including also a control population are warranted ^33^. Patients at risk should of course be assessed prior to surgery/anaesthesia. Optimal screening method is however not well defined ^11^.

There are several limitations with our study. It is merely an observational “explorative” study, and we could only include 23 mothers and gained registration form 20. We had a high number of mothers that declined to participate after having been informed about the monitoring techniques. The portable polygraphy is intended for use in the home for sleep apnoea screening instead of being in-hospital for a full polysomnography. The equipment involves straps around the thorax and abdomen, nasal prongs and a pulseoximetry probe all connected with cables to the monitoring unit. We included mothers with a BMI between 30 and 42 (mean 35) and none of our mothers had a known sleep apnoea. Merely three had an ESS of more than 10. Higher BMI and higher number of patients, possibly with more signs and symptoms of sleep apnoea would have been of interest. We are not able to assess sleep time, whether the mothers studied were asleep or merely rested. A full polysomnography would be needed for further in depth analysis. One may however strongly question whether that is ethical in a mothers’ first night after caesarean section. The number of mothers studied is low and it is indeed not possible to make any firm statistical assessments based on the sparse data available.

In conclusion, we found in this explorative study that portable polygraphy is cumbersome and many mothers decline its use. It seems also reasonable to conclude that although episodes of oxygen saturation decrease were not infrequently noticed, upper airway collapse, obstructive hypo/apnoea, role as risk factor for respiratory depression during the first night after caesarean section in spinal anaesthesia with addition of low dose intrathecal morphine even in obese mothers was not commonly seen. However, further studies with a combination of RR monitoring, TcCO _2_ monitor and SpO _2_ seems warranted, especially in high risk mothers. Studies focused on preoperative screening with night time respiratory polygraphy in obese patients, at risk for sleep breathing disorder, are also warranted.

## Data availability

The data referenced by this article are under copyright with the following copyright statement: Copyright: © 2018 Hein A and Jakobsson JG

Data associated with the article are available under the terms of the Creative Commons Zero "No rights reserved" data waiver (CC0 1.0 Public domain dedication). 

Dataset 1: Raw data for the study polygraphy on the first night after caesarean section in spinal anaesthesia with morphine in obese mothers by Hein *et al.* The three patients who were excluded from analysis are highlighted. doi, 10.5256/f1000research.13206.d185388 ^34^

## Footnotes

http://epworthsleepinessscale.com/about-the-ess/
