# Supplementary material for: Portable respiratory polygraphy monitoring of obese mothers the first night after caesarean section with bupivacaine/morphine/fentanyl spinal anaesthesia
Source: F1000Res. 2018 Feb 7;6:2062. Originally published 2017 Nov 29. [Version 2] doi: 10.12688/f1000research.13206.2 (PMC5820605; doi:10.12688/f1000research.13206.2)
Supplement: Peer review history

# Peer review: Referee response for version 2

**DOI:** 10.5256/f1000research.15048.r30645

**Published:** 2018-02-19

**Authors:** Patrick Thorton, Sunil Kumar Chauhan

**Affiliations:** 1 Rotunda Hospital, Dublin, Ireland

Thank you author for addressing our comments and concerns about your study. The article gives appropriate insight of methodology and challenges you had while recruiting pregnant mothers for monitoring in post op period. It was well organised study, but with small sample size.

We have read this submission. We believe that we have an appropriate level of expertise to confirm that it is of an acceptable scientific standard.

# Peer review: Referee response for version 2

**DOI:** 10.5256/f1000research.15048.r30644

**Published:** 2018-02-12

**Authors:** Lone Nikolajsen, Nina Stockfleth Buch

**Affiliations:** 1 Department of Anesthesiology and Intensive Care, Aarhus University Hospital, Aarhus, Denmark

The article has improved following revision. Did the authors use any statistical analysis to study the possible correlation between ODI and AHI? (Spearmans rho?).

 It could still benefit from shortening (discussion) and a revision of the English language.

We have read this submission. We believe that we have an appropriate level of expertise to confirm that it is of an acceptable scientific standard.

# Peer review: Referee response for version 1

**DOI:** 10.5256/f1000research.14329.r28494

**Published:** 2018-01-26

**Authors:** Patrick Thorton, Sunil Kumar Chauhan

**Affiliations:** 1 Rotunda Hospital, Dublin, Ireland

INTRODUCTION

 The aim of this study was to assess monitoring capabilities of portable respiratory polygraph device in detecting respiratory depression due to intrathecal morphine in parturients for lower segment caesarean section. Although the number of factors causing respiratory depression is not just limited to intrathecal morphine, obesity, residual effect of anaesthesia, immobilisation and abdominal surgery itself are contributors for respiratory depression. The device used for the study seems good for diagnosing respiratory depression in community for patients with OSA, but its usefulness in peri-operative setting is still to be proved.

 Comments:

- This study done on exploring the optimal monitoring device to diagnose respiratory depression with intrathecal morphine for LSCS in high BMI parturients, does not rule out undiagnosed OSA patients before abdominal surgery esp. when the patients were enrolled during the first antenatal visit and since that time till surgery patient had undergone changes in their BMI and usual respiratory physiological changes.
- The device respirator is not adequately described esp. it’s functioning as well it’s methodology to diagnose respiratory depression is seriously questionable as it fails to show basic correlation between AHI and ODI.
- The study design is prospective and observational, but would be of more use if it has compared with routine monitoring in subset of patients with intrathecal morphine for LSCS, to give idea of usefulness of the device.
- Mean bedtime during which monitoring was done was never described as whether patient was asleep or was merely resting, which could seriously alter the results.
- There was no mention of type of breakthrough analgesia (opioid or non opioid) used postoperatively which could alter the results.
- The sample size to comment on correlation between AHI and ODI seems quite small to be conclusive and reproducible.
- The results showed comparison with routine monitoring but data in support of routine monitoring was not provided.
- Again high AHI and non reproducible ODI raises serious concerns about sensitivity of portable respirator.
- As far as studies statistical significance I have limited knowledge for which input from statistician would be highly recommended.
- I agree with the conclusion by authors that device is cumbersome with no reproducible results until proven otherwise by studying with large sample size in patients with preoperative screening in obese parturients (with already decreased respiratory reserve ) at risk of OSA and comparing with routine monitoring.

Discussion:

  

- This study has some minor limitations in view of enrollments during first antenatal visit It would be better if more clarity with data was shown for duration of sleep rather than rest.
- Post operative analgesia for breakthrough pain needs to be explained.
- Any study to quote for the efficacy of device would be of great value before using it in peri-operative setting.
- Any comorbidity like PIH or chest infections before surgery or any major fluid shift with any major blood losswas not ruled out or if it was then not declared in the study design which may easily alter the results.
- Post operative analgesia for breakthrough pain needs to be explained.
- Any study to quote for the efficacy of device would be of great value before using it in peri-opera- tive setting.

We have read this submission. We believe that we have an appropriate level of expertise to confirm that it is of an acceptable scientific standard, however we have significant reservations, as outlined above.

# Response

**Published:** 2018-01-26

**Authors:** Jan Jakobsson

**Affiliations:** Department of Physiology and Pharmacology, Karolinska Institutet, Sweden

***Dear Referee, thank you for important and adequate comments and criticism. The study is small and “explorative” – we still feel that it is of importance to present the available findings in structured fashion. We have tried to address all your comments and queries in Version 2 that has just been uploaded in the manuscript management system.***

Comments:

This study done on exploring the optimal monitoring device to diagnose respiratory depression with intrathecal morphine for LSCS in high BMI parturients, does not rule out undiagnosed OSA patients before abdominal surgery esp. when the patients were enrolled during the first antenatal visit and since that time till surgery patient had undergone changes in their BMI and usual respiratory physiological changes.

***We have in version 2 put strong emphasis on the need for further studies in pregnant women per se and especially in obese mothers. Sleep apnea should be considered and assessed for.***

**

The device respirator is not adequately described esp. it’s functioning as well it’s methodology to diagnose respiratory depression is seriously questionable as it fails to show basic correlation between AHI and ODI.

***We did use a standard ambulatory monitor, the Embletta and the NOx and subsequent the *ResMed Sweden AB, Kista, Sweden *analysis program with manual screening. The accuracy in the program has been assessed as adequate by the Swedish Agency for Health Technology Assessment and Assessment of Social Services. We did see a discrepancy between ODI and AHI, the exact reason for this is to us unclear. The group may have had desaturation episodes due to low respiratory rate, atelectasis and thus alveolar hypoventilation, but we cannot exclude a monitoring error.***

The study design is prospective and observational, but would be of more use if it has compared with routine monitoring in subset of patients with intrathecal morphine for LSCS, to give idea of usefulness of the device.

***Agree, this would be of interest for further studies, but considering the cumbersomeness of the polygraphy I doubt it is worth the effort. I would much prefer preopstudies trying to find patients at risk during pregnancy. We have expanded on this is abstract, introduction and discussion.***

**

Mean bedtime during which monitoring was done was never described as whether patient was asleep or was merely resting, which could seriously alter the results.

***Indeed; this is also one of limitations, we do not have any objective measure of real sleep time, merely 1 ^st^ night registration.***

There was no mention of type of breakthrough analgesia (opioid or non opioid) used postoperatively which could alter the results.

***The mothers provided rescue analgesics preferentially buprenorphine is added. All mothers had paracetamol regular prescribed.***

The sample size to comment on correlation between AHI and ODI seems quite small to be conclusive and reproducible.

***We have purposely not made any statistical assessment of AHI and OFI findings. This is a explorative observational study and we fully agree there is not data to make any firm statistical conclusions***

The results showed comparison with routine monitoring but data in support of routine monitoring was not provided.

Again high AHI and non reproducible ODI raises serious concerns about sensitivity of portable respirator.

***We cannot give any firm explanation to the discrepancy observed. We have further commented on this finding and we feel that there may be patient factors to possibly explain the finding and there are other studies also discussing discrepancy between ODI/AHI. **Novel parameters for evaluating severity of sleep disordered breathing and for supporting diagnosis of sleep apnea-hypopnea syndrome.**Kulkas A, Tiihonen P, Eskola K, Julkunen P, Mervaala E, Töyräs J. J Med Eng Technol. 2013 Feb;37(2):135-43.***

As far as studies statistical significance I have limited knowledge for which input from statistician would be highly recommended.

***We have purposely not made any statistical assessment of AHI and OFI findings. This is a explorative observational study and we fully agree there is not data to make any firm statistical conclusions***

I agree with the conclusion by authors that device is cumbersome with no reproducible results until proven otherwise by studying with large sample size in patients with preoperative screening in obese parturients (with already decreased respiratory reserve ) at risk of OSA and comparing with routine monitoring.

Discussion:

This study has some minor limitations in view of enrollments during first antenatal visit It would be better if more clarity with data was shown for duration of sleep rather than rest.

***Extensively commented in version 2***

Post operative analgesia for breakthrough pain needs to be explained.

***Added in version 2***

Any study to quote for the efficacy of device would be of great value before using it in peri-operative setting.

***Added in the Method section***

Any comorbidity like PIH or chest infections before surgery or any major fluid shift with any major blood loss was not ruled out or if it was then not declared in the study design which may easily alter the results.

***Mothers were considered “healthy” no obvious signs of infection or fluid overload was observed.***

Post operative analgesia for breakthrough pain needs to be explained.

***Added***

Any study to quote for the efficacy of device would be of great value before using it in peri-opera- tive setting.

**On behalf of the authors Jan G Jakobsson**

# Peer review: Referee response for version 1

**DOI:** 10.5256/f1000research.14329.r28813

**Published:** 2018-01-19

**Authors:** Lone Nikolajsen, Nina Stockfleth Buch

**Affiliations:** 1 Department of Anesthesiology and Intensive Care, Aarhus University Hospital, Aarhus, Denmark

This observational study by Hein and Jacobsen examines the use of a portable respiratory polygraphy device for monitoring of obese mothers for respiratory depression the first night after caesarean section performed with bupivacaine/morphine/fentanyl spinal anaesthesia. The topic is interesting and relevant, as an increasing number of parturients is expected to present with high antenatal BMI in the future. A total of 20 women were monitored the first night after caesarean section. The authors conclude that the use of the polygraphy device is cumbersome and that obstructive events and episodes of desaturation were not commonly synchronized.

 Comments:

 Abstract: For the reader not familiar with this technique or the apnoe/hypopnea index (AHI), it is very difficult to interpret the results, e.g. AHI values of 5-15? And what does an ODI of 4.4 mean?

 For these reasons, the abstract is simply difficult to read.

 The introduction is a bit too long. The authors should consider omitting e.g. the sentence starting with Dahan in the first paragraph, and the second paragraph on definition of respiratory depression.

 Methods and results: Parts of the text could be written more clearly, e.g. page 4 top: Additive medication to treat side effects, such as pain..??? Is pain a side effect?

 The description of AHI, ODI etc. is very technical. Again, it is difficult for the reader not familiar with the technique, to interpret the values and relate them to the clinical setting. What is the clinical relevance of mild AHI/ ODI, mean nadir SpO _2_, mean and max TcCO _2_? The same applies to the ESS score: is a score of e.g. 5 good or bad? 

 Did the women receive opioids during the study period? (Respiratory depression could be caused by ex. oral opioids).

 Discussion: Could be shortened.

 Figure 2 is difficult to read.

 Could some of the results be presented in a table?

 The high number of abbreviations makes the paper more difficult to read.

 The cohort described/studied by Subramani *et al*. (reference 20) might not be comparable with the group studied in this paper.

 In summary, a nice paper on an interesting and relevant topic but it would benefit from a revision.

 Review performed by MD Nina Stockfleth Buch, Department of Anesthesiology, Aarhus University Hospital, Denmark (approved by professor Lone Nikolajsen, Department of Anesthesiology, Aarhus University Hospital, Denmark).

We have read this submission. We believe that we have an appropriate level of expertise to confirm that it is of an acceptable scientific standard, however we have significant reservations, as outlined above.

# Response

**Published:** 2018-01-22

**Authors:** Jan Jakobsson

**Affiliations:** Department of Physiology and Pharmacology, Karolinska Institutet, Sweden

Comments to reviewers at Department of Anesthesiology, Aarhus University Hospital, Denmark).

Thank you for effective review of our paper and important comments and remarks. We used the America Academy of Sleep Medicine definitions, that the apnea/hypopnea index, the number of apnea/hypopnea events registered per hour are graded into three categories of Obstructive Sleep Apnea (OAS): Mild OSA: AHI of 5-15, Moderate OSA: AHI of 15-30 and Severe OSA: AHI of more than 30. Zero to 4 episodes, and AHI of<5 is commonly assessed as *normal*. According to the 2007 guidelines from the American Academy of Sleep Medicine, any event with a 3 percent drop in blood oxygen levels is counted as an oxygen desaturation, and the index is the number of desaturation episodes per hours. The screening and assessment for sleep apnea is updated in JAMA 2017 and criteria have not changed. The equipment software is screening for both the AHI and ODI. The program also provide each desaturation episode minimum blood oxygen saturation SpO2 level measured, the oxygen desaturation episode *nadir*.

We consider our findings as signs of normal/mild breathing pattern; all but 2 mothers showed an AHI well within normal and mild ranges and 2 had values in the moderate zone (15.3 and 18.2, thus in the lower edge of moderate 15-39). The apnea/hypopenea episodes had duration of 11 to 111 seconds with a lowest SpO2 60 mean 73 % (60 – 81).

None of the mothers showed a transcutaneous reading of above 5 kPa, thus none of our monitored mothers had a transcutaneous CO2 above normal.

The questionnaires used ESS for screening has been the standard tool at our department. We used the recommended grading;

0-5 Lower Normal Daytime Sleepiness

6-10 Higher Normal Daytime Sleepiness

11-12 Mild Excessive Daytime Sleepiness

13-15 Moderate Excessive Daytime Sleepiness

16-24 Severe Excessive Daytime Sleepiness

Also the ESS scores were low 3 mothers scored more than 10 commonly assessed as “threshold value”.  

The description of pain and additional medication should be revised, to read easier.

We provide our assessment of the “seemingly normal findings” from the polysomnography recordings in the first sentences of the discussion. We strongly believe that our study show that polysomnography is cumbersome and complex to use postoperatively and does not add valuable information. It is indeed intended for assessment of sleep apnea and is not primarily a monitoring device, it include no alarms to alert in case of major deviation it merely records and compile data for post recording assessment. Respiratory rate, SpO2 and CO2 monitoring seems of much more value. **Preoperative assessment** should of course be performed eventually including polysomnography in at risk patient . If positive findings CPAP should be recommended and used in conjunction to surgery/anesthesia.

We will make a version 2 as soon as we have and additional referee report.

References

https://aasm.org/resources/factsheets/sleepapnea.pdf

http://epworthsleepinessscale.com/about-the-ess/

Jonas DE, Amick HR, Feltner C, Weber RP, Arvanitis M, Stine A, Lux L, Harris RP. Screening for Obstructive Sleep Apnea in Adults: Evidence Report and Systematic Review for the US Preventive Services Task Force. JAMA. 2017 Jan 24;317(4):415-433.

Swedish Council on Health Technology Assessment. Obstructive Sleep Apnoea Syndrome: A Systematic Literature Review [Internet]. Stockholm: Swedish Council on Health Technology Assessment (SBU); 2007 Jun.

Collop NA, Anderson WM, Boehlecke B, Claman D, Goldberg R, Gottlieb DJ, Hudgel D, Sateia M, Schwab R; Portable Monitoring Task Force of the American Academy of Sleep Medicine. Clinical guidelines for the use of unattended portable monitors in the diagnosis of obstructive sleep apnea in adult patients. Portable Monitoring Task Force of the American Academy of Sleep Medicine. J Clin Sleep Med. 2007 Dec 15;3(7):737-47.

Madhusudan P, Wong J, Prasad A, Sadeghian E, Chung FF. An update on **preoperative** **assessment** and preparation of surgical patients with obstructive **sleep apnea**. Curr Opin Anaesthesiol. 2018 Feb;31(1):89-95.

Roesslein M, Chung F. Obstructive **sleep apnoea** in adults: peri-operative considerations: A narrative review. Eur J Anaesthesiol. 2018 Jan 2. doi: 10.1097/EJA.0000000000000765. [Epub ahead of print]
